# Photopyroelectric Spectroscopic Studies of ZnO-MnO_2_-Co_3_O_4_-V_2_O_5_ Ceramics

**DOI:** 10.3390/ijms12031625

**Published:** 2011-03-03

**Authors:** Zahid Rizwan, Azmi Zakaria, Mohd Sabri Mohd Ghazali

**Affiliations:** 1 Department of Physics, Faculty of Science, University Putra Malaysia, 43400 UPM Serdang, Selangor, Malaysia; E-Mails: zahidrizwan64@gmail.com (Z.R.); mgm.sabri@gmail.com (M.S.M.G.); 2 Advanced Materials and Nanotechnology Laboratory, Institute of Advanced Technology, University Putra Malaysia, 43400 UPM Serdang, Selangor, Malaysia

**Keywords:** photopyroelectric spectroscopy, ZnO, V_2_O_5_, sintering, secondary phases, optical energy band-gap

## Abstract

Photopyroelectric (PPE) spectroscopy is a nondestructive tool that is used to study the optical properties of the ceramics (ZnO + 0.4MnO_2_ + 0.4Co_3_O_4_ + xV_2_O_5_), x = 0–1 mol%. Wavelength of incident light, modulated at 10 Hz, was in the range of 300–800 nm. PPE spectrum with reference to the doping level and sintering temperature is discussed. Optical energy band-gap (*E_g_*) was 2.11 eV for 0.3 mol% V_2_O_5_ at a sintering temperature of 1025 °C as determined from the plot (*ρhυ*)^2^ *versus* *hυ*. With a further increase in V_2_O_5_, the value of *E_g_* was found to be 2.59 eV. Steepness factor ‘*σ*_A_’ and ‘*σ*_B_’, which characterize the slope of exponential optical absorption, is discussed with reference to the variation of *E_g_*. XRD, SEM and EDAX are also used for characterization of the ceramic. For this ceramic, the maximum relative density and grain size was observed to be 91.8% and 9.5 μm, respectively.

## 1. Introduction

ZnO based ceramic semiconductors are widely used as gas sensors [1], piezoelectrics, electrodes for solar cells, phosphors, transparent conducting films and varistors [2]. Varistors possess high energy absorption capability against various surges. They are extensively used as protective devices to regulate transient voltage surges of unwanted magnitudes due to their fast response to over voltage transients. They sense and clamp transient voltage pulses in nanosecond speed [3]. The exact role of many additives in the electronic structure of ZnO based varistors is uncertain. Improving the electrical properties of ZnO based varistors is under research. The formulation of varistors that have high non-linear characteristics is the most important parameter to consider. Varistors are formed with small amounts of other metal oxides such as Al_2_O_3_, Bi_2_O_3_, Co_3_O_4_, Cr_2_O_3_, MnO, Sb_2_O_3_, TiO_2_, *etc*. Such additives are the main tools that are used to improve the non-linear response and stability of the varistor [4]. This nonlinear response can be explained by the mechanism concerning the grain boundaries and associated defect concentration gradients [5]. Electrical properties of the ceramic ZnO depend on the distribution of vacancies, impurities and their behavior. Much has been done in *I–V* characterization of ZnO based varistors in previous studies [4]. It is essential to obtain information on the optical absorption behavior of the ceramic ZnO doped with metal oxides for the examination of electronic states. The optical absorption behavior of ZnO doped with MnO_2_, Co_3_O_4_ is discussed for the different doping levels of V_2_O_5_ at different processing conditions.

## 2. Experimental Section

ZnO (99.9% pure, Alfa Aesar) was doped with 0.4MnO_2_ (99.999% pure, Alfa Aesar), 0.4Co_2_O_3_ (99.7% pure, Alfa Aesar) and xV_2_O_5_ (99.6% pure, Alfa Aesar) where x = 0–1.5 mol%. The detail of the composition is given in Table 1. Powder of all ingredients (24 hour ball milled) of each mole percent was pre-sintered at 700 °C for 90 minutes in open atmosphere at a heating and cooling rate of 5 °C/min. Samples were ground and polyvinyl alcohol (1.1 wt %) was added as a binder. The dried powder was pressed under a force of 800 kg cm^−2^ to form a disk of 10 mm diameter. Finally the disks were sintered at 950 and 1025 °C for 2 hours in air at a heating and cooling rate of 4 °C min^−1^. The disk from each sample was ground for 1 hour and granulated by sieving through a 75-mesh screen for the photopyroelectric (PPE) spectroscopy and XRD analysis. Density was calculated using geometrical method. Polished samples were thermally etched for microstructure analysis. Grain size was determined by the grain boundary crossing method. Cu K_α_ radiation with PANAalytical (Philips) X’Pert Pro PW1830 was used for X-ray analysis. XRD data were analyzed by X’Pert High Score software.

The measurement of PPE signal amplitude has been described elsewhere [6]. A light beam (300 to 800 nm) from one kW Xenon arc lamp, mechanically chopped at 10 Hz was used for PPE measurements. Optical absorption coefficient (*β*) varies with the excitation photon energy (*hυ*) [7]. It is given by the expression, (*βhυ*)^2^ = (*hυ* *− E_g_*), where *hυ* is the photon energy, *C* is constant and *E*_g_ is the optical energy band gap. PPE signal intensity (*ρ*) is directly proportional to *β*, hence (*ρhυ*)^2^ is related to *hυ* linearly. From the plot of (*ρhυ*)^2^ *versus* *hυ*, *E*_g_ is obtained by extrapolating the linear fitted region to zero.

Optical absorption edge has been observed in a variety of crystalline and amorphous materials. The optical-absorption edge has an important role in electron or exciton-phonon interactions [8]. It is found that PPE signal intensities plotted semi logarithmically vary linearly with the photon energy just lower than the fundamental absorption edge [9]. Therefore, an empirical relation for absolute measuring temperature (*T*) and photon energy (*hυ*) is given by the equation:

(1) $$P=P_{0}e\left(\frac{\sigma (h\upsilon -h\upsilon_{0}}{kT}\right)$$

where *k* is the Boltzmann constant and *P*_0_, *σ*, *υ*_0_ are fitting parameters [10,11]. The value *σ/kT* determines the exponential slope, where *σ* is the steepness factor and is characterized in optical absorption edge. The steepness factor is found (*σ*_A_ in region-A and *σ*_B_ in region-B) from the PPE spectrum.

## 3. Results and Discussion

**Figure 1.**
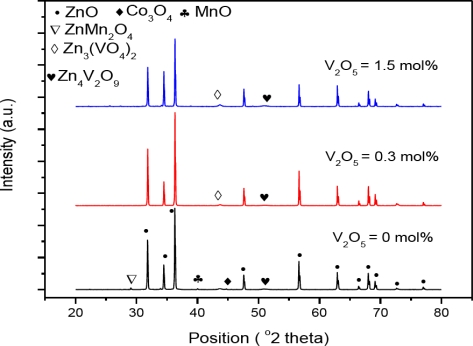
XRD patterns of V_2_O_5_ doped ZnO at 1025 °C.

The XRD pattern of the V_2_O_5_ doped ZnO for the sintering temperature of 1025 °C can be seen in Figure 1. The samples at 0 mol% of V_2_O_5_ contain small peaks related to Co_3_O_4_ (reference code 00-042-1467) at both sintering temperatures but peaks are clearer at 1025 °C. Very small peaks related to ZnMn_2_O_4_ (reference code 01-077-0470) were also found at both sintering temperatures. Samples doped at 0.3 mol% V_2_O_5_ contain the secondary phases Zn_3_(VO_4_)_2_ (reference code 00-034-0378), Zn_4_V_2_O_9_ (reference code 01-077-1757). The same phases are also found at all higher doping levels of V_2_O_5_.

**Figure 2.**
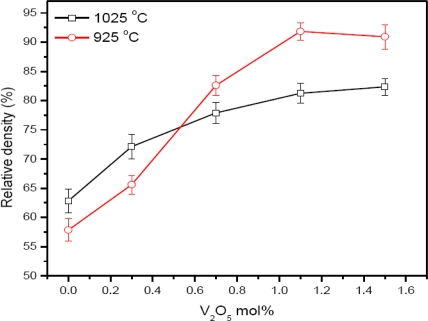
Density variation with V_2_O_5_ doping level.

The density increases from 57.8 to 91.8% of the theoretical density at a sintering temperature of 925 °C for a 2 hour sintering time, Figure 2. The density increases with the increase of V_2_O_5_ mol% and is in accordance with the literature [12]. The density increase at 925 °C above 0.7 mol% of V_2_O_5_ indicates that the densification process is essentially completed at the sintering temperature above 900 °C [12]. It is expected that the vanadium-rich liquid phase Zn_3_(VO_4_)_2_ enhances the densification by a solution and re-precipitation of ZnO [13]. The density of the ceramic is increased from 62.8 to 82.4% for the sintering temperature of 1025 °C. Density increases slowly compared to at the lower sintering temperature. The density has a lower value above 0.7 mol% of V_2_O_5_ at a sintering temperature of 1025 °C than 925 °C. This lower density may be due to the volatility of V_2_O_5_ [13].

**Figure 3.**
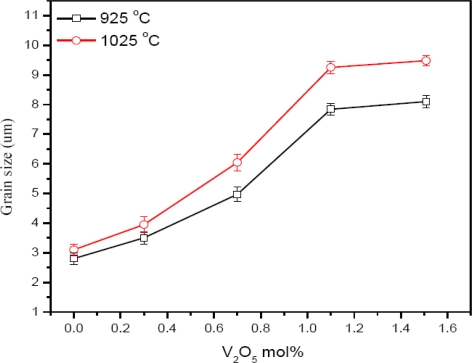
Grain growth behavior of the sample doped with V_2_O_5_.

**Figure 4.**
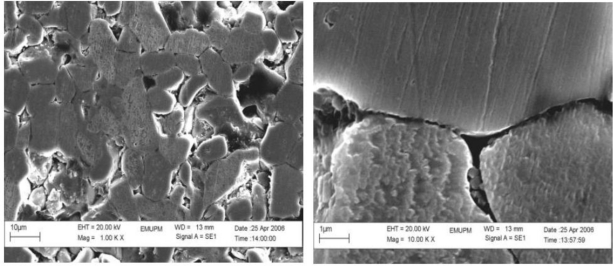
SEM images for the ceramic sintered with 1.5 mol% of V_2_O_5_ at 1025 °C for 2 hours. Two examples are shown.

**Figure 5.**
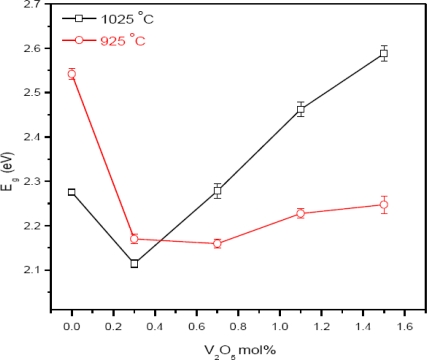
Dependence of *E_g_* on V_2_O_5_ mol%.

Examination of the microstructure, Figure 3, shows that the grain size of the ceramic at 0 mol% of V_2_O_5_ is 2.8 and 3.1 μm and is increased to 8.1 and 9.48 μm at the sintering temperature of 925 and 1025 °C, respectively. The grain size is increased with the increase of sintering temperature at all mol% of V_2_O_5_. Large grains have oblong shape and small grains are also found in the ceramic. Exaggerated ZnO grain growth is found in the samples, Figure 4. This is due to the high reactivity of the V-rich liquid phase during sintering, which causes abnormal grain growth [14]. The addition of V_2_O_5_ can enhance the densification and grain growth behavior. This fact can be attributed to the formation of Zn_3_(VO_4_)_2_, which acts as a liquid phase sintering aid [12]. EDX analysis shows that the vanadium is distributed at the grain boundaries as well as triple point junction [15]. Co and Mn are distributed in the grain boundaries and in the grain interiors [16]. The value of *E_g_* is reduced from 3.2 eV (pure ZnO) to 2.28 and 2.54 eV at 0 mol% of V_2_O_5_ for the sintering temperatures of 1025 and 925 °C, respectively (Figure 5). This is due to 0.4 mol% of MnO_2_ and Co_3_O_4_ because the reduction of *E_g_* is due to the introduction of interface states by Mn and Co ions as the ionic radius of Co and Mn is smaller than that of Zn^2+^. With the addition of 0.3 mol% of V_2_O_5_, the *E_g_* decreases from 2.28 and 2.54 eV to 2.17 and 2.11 eV at 1025 and 925 °C, respectively. It is due to the introduction of the interface states.

**Figure 6.**
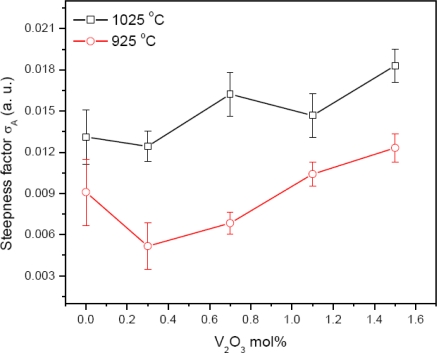
Variation of *σ*_A_ with V_2_O_5_ mol%.

The ionic radius of Zn^2+^ is 0.74 Å and the ionic radius of Vanadium is 0.59 Å, so the reduction in the value of *E_g_* at 0.3 mol% of V_2_O_5_ is due to the limited substitution of Vanadium ions in the ZnO lattice. The value of *E_g_* is increased with the doping level of V_2_O_5_ beyond 0.3 mol%. It is expected that this may be due to the segregation of the V_2_O_5_ forming secondary phases Zn_3_(VO_4_)_2_ and Zn_4_V_2_O_9_ and reduces the interface states. The further increase in the value of *E_g_* may be due to the high volatility of V_2_O_5_ at the high sintering temperature of 1025 °C. The steepness factor *σ*_A_, Figure 6, increased with the increase of V_2_O_5_ doping level for the both sintering temperatures 925 and 1025 °C for the 2 hour sintering time. The increase in the value of *σ*_A_ with the doping level indicates the decrease in the PPE signal intensity. The decrease in the PPE signal intensity corresponds to the decrease in structural disordering. This indicates the decrease in the interface states with the doping level of V_2_O_5_. Resultantly, the value of *E_g_* increases slightly as shown in Figure 5.

Generally an exponential tail (in region-B) for crystalline semiconductors can be characterized by:

(2) $${\left(\sigma_{\text{B}}/KT\right)}^{-1}=A<{U^{2}>}_{T}/C_{o}$$

*C_o_* is the exponential tail parameter of the order of unity and <*U*^2^>*_T_* is the thermal average of the square of the displacement of the atoms from their equilibrium positions. The term <*U*^2^>*_T_* expresses the energy of displacement of atoms [17,18].

**Figure 7.**
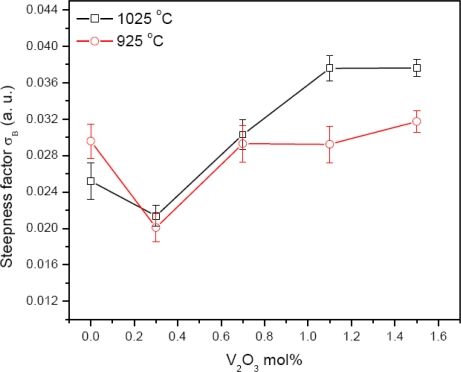
Variation of *σ*_B_ with V_2_O_5_ mol%.

The value of the steepness factor (*σ*_B_) decreases with the increase of doping of V_2_O_5_ for sintering temperatures of 925 and 1025 °C, Figure 7. This indicates that the average thermal displacement energy of atoms is increasing. This increase indicates an increase in structural disordering. Thus, the value of *E_g_* decreases. Above 0.3 mol%, the value of *σ*_B_ increases with the increase of V_2_O_5_ for both sintering temperatures of 925 and 1025 °C. This indicates that the average thermal displacement energy of atoms is decreasing, which indicates a decrease in structural disordering. Correspondingly, the value of *E_g_* increases. This may be due to the high volatility of the V_2_O_5_ and the secondary phases developed in the ceramics.

## 4. Conclusions

XRD results confirm the hexagonal phase of ZnO and few peaks of the secondary phases ZnMn_2_O_4_, Zn_3_(VO_4_)_2_ and Zn_4_V_2_O_9_. The EDX analysis shows that the V, Co and Mn are distributed at the grain boundaries and grain interiors. The maximum and minimum grain size is found to be 9.48 μm for 1025 °C and 2.8 μm for the 925 °C sintering temperature, respectively. The maximum and minimum relative density is found to be 82.4 for 1025 °C and 57.9 for 925 °C sintering temperature, respectively. The optical energy band-gap is reduced to 2.11 eV for 0.3 mol% V_2_O_5_ at the sintering temperature of 1025 °C. PPE spectrometry proved to be a useful tool to study the optical absorption behavior along with the other electrical measurements of ZnO based varistors.

## Figures and Tables

**Table 1.** Composition of each ceramic sample.

**S #**	**ZnO (mol%)**	**MnO_2_ (mol%)**	**Co_3_O_4_ (mol%)**	**V_2_O_5_ (mol%)**
1	99.2	0.4	0.4	0
2	98.9	0.4	0.4	0.3
3	98.5	0.4	0.4	0.7
4	98.1	0.4	0.4	1.1
5	97.7	0.4	0.4	1.5
